# Research on a Polyolefin Composite Modifier for High-Temperature and Heavy-Duty Pavement and Performance of Its Modified Asphalt

**DOI:** 10.3390/polym18010026

**Published:** 2025-12-22

**Authors:** Runduo Ding, Xianhe Wang, Wei Wang, Haoran Wang, Huaxin Chen, Yanjun Zhang

**Affiliations:** 1 School of Highway, Chang’an University, Xi’an 710061, China; 2 China Highway (Beijing) Engineering Materials Technology Co., Ltd., Beijing 100088, China

**Keywords:** polyolefin, composite modified asphalt, compatibility, high-temperature performance, self-healing capacity, modification mechanism

## Abstract

To address the challenges of rutting and performance balance in asphalt pavements under high-temperature and heavy-load conditions, a novel polyolefin composite modifier (PCM-H) was developed from waste tire rubber powder, recycled ethylene vinyl acetate (EVA), acrylonitrile butadiene styrene (ABS), petroleum resin, and polymer additives. The chemical characteristics, thermal stability, and compatibility mechanisms of PCM-H were compared with those of two commercial modifiers (PCM-1 and PCM-2) using Fourier transform infrared spectroscopy (FTIR), thermogravimetric analysis (TGA), and differential scanning calorimetry (DSC). PCM-H exhibited superior compatibility and thermal stability. In contrast, PCM-2 tends to crystallize and precipitate within the 180–200 °C range, which is detrimental to the stability of the composite system. At an optimal dosage of 10 wt% in styrene–butadiene–styrene (SBS) modified asphalt, PCM-H formed a uniform dispersion and, through crosslinking reactions, established a three-dimensional network structure. Subsequently, the performance of composite modified asphalts, prepared with each of the three modifiers at their respective optimal dosages, was evaluated comparatively. Performance evaluations demonstrated that all polyolefin-modified asphalts significantly outperformed the conventional SBS modified asphalt. The PCM-H modified asphalt (PCM-H MA) exhibited the most superior performance, achieving a performance grade (PG) exceeding 94 °C, along with exceptional high-temperature elasticity and creep resistance, superior low-temperature cracking resistance, and enhanced fatigue healing capability. The results indicated that the crosslinked network structure effectively enhances asphalt cohesion, thereby providing a synergistic improvement in both high- and low-temperature performance. This study provides an effective solution and theoretical basis for developing high-performance pavement materials resistant to high temperatures and heavy loads conditions.

## 1. Introduction

The rapid expansion of highway transportation has led to a consistent annual increase in traffic volume, with heavy loads and overloading becoming increasingly prevalent. Consequently, existing asphalt pavements often fail to withstand the demands of heavy-load traffic throughout the intended design life, exhibiting premature distress such as rutting and cracking. Furthermore, the Sixth Assessment Report (AR6) of the Intergovernmental Panel on Climate Change (IPCC) indicates that, under ongoing climate change, the intensity, frequency, and spatial extent of extreme heatwaves across most land areas are increasing [1,2,3]. The combination of frequent extreme high temperatures and heavy traffic loads accelerates the deterioration of asphalt pavements. This synergy significantly increases the susceptibility to permanent deformation (e.g., rutting) and other structural failures, leading to a significant reduction in service life [4]. These distress conditions not only elevate road maintenance costs but also pose a significant threat to driving safety. The synergistic effect of high temperatures and heavy loads leads to significant performance deficiencies in traditional asphalt pavements. First, at elevated temperatures, the modulus and shear resistance of asphalt materials decrease, rendering them prone to rutting under heavy-duty vehicle loads. Second, in low-temperature environments, asphalt becomes brittle and susceptible to thermal, transverse, and fatigue cracking under repeated traffic loading [5]. A global research consensus holds that heavy-traffic-induced damage constitutes a critical challenge in asphalt pavement design. For decades, research in developed countries has prioritized pavement distress that manifests within the design life, particularly early-stage damage in heavy-duty pavements, including transverse and longitudinal cracking, fatigue cracking (alligator cracking), rutting, raveling, settlement, and potholes [6,7]. Consequently, simultaneously enhancing the high-temperature stability and low-temperature crack resistance of asphalt materials has emerged as a critical and urgent technical challenge in road engineering.

The application of modifiers represents an effective approach for enhancing the performance of asphalt pavement. However, most commercial asphalt modifiers currently available on the market exhibit certain limitations and challenges in practical applications.

Although traditional anti-rutting agents and high-modulus agents can enhance the high-temperature stability of asphalt mixtures, their functionality is limited and often compromises low-temperature performance. This makes it challenging to meet the dual high- and low-temperature requirements for asphalt pavement in regions with extreme seasonal variations. Research has demonstrated that although certain high-modulus agents increase the modulus and high-temperature deformation resistance of asphalt, they reduce the low-temperature crack resistance by 15–20% [8]. High viscosity and high elasticity asphalt can offer a more balanced performance profile, improving high- and low-temperature properties alongside fatigue resistance. However, these blends typically require a high styrene–butadiene–styrene (SBS) content, resulting in elevated materials costs. For example, Yang et al. [9] prepared high viscosity elastic anti-fatigue agent asphalt using basic asphalt, SBS, active desulfurized rubber, and aromatic oil. The optimal dosages of SBS and active desulfurized rubber are 3.5 wt% −4.5 wt% and 22 wt% −25 wt%, respectively. Similarly, Xia et al. [10] utilized SBS, tackifiers, and solubilizers as primary raw materials to determine the optimal composition for a high viscosity asphalt via a three-factor three-level orthogonal experiment design. 

In recent years, polyolefin modifiers have garnered significant attention due to their excellent mechanical properties and cost-effectiveness. Specifically, modifiers derived from recycled plastics offer the dual advantages of enhancing asphalt performance while promoting environmental protection and resource reuse [11,12,13,14,15]. Consequently, the preparation of modifiers from discarded polyolefin plastics has become a prominent research focus, offering a viable solution to plastic pollution and a potential reduction in material costs [16,17,18]. Two primary methods exist for incorporating waste polyolefin plastics into asphalt concrete: the “dry” method and the “wet” method. In the dry method, a plastic modifier is directly mixed with aggregate and asphalt prior to paving. In the wet method, the plastic modifier is first blended with asphalt to create a homogeneous modified asphalt binder, which is subsequently mixed with aggregate [19,20]. For instance, Ren et al. [21] employed the wet method, incorporating waste polyethylene as a modifier at concentrations of 5, 7, and 9 wt% relative to the base asphalt mass. Their protocol involved adding the waste plastic particles proportionally to 165 °C base asphalt, controlling the temperature between 160 and 170 °C, and mixing at low speed for 30 min. After complete melting, the blend was placed in a 120 °C oven for 15–20 min. This was followed by high-speed shearing for at least half an hour to produce the final modified asphalt, whose performance indicators were then evaluated. The results indicated that waste plastics could significantly improve the high-temperature stability of both the asphalt binder and the resultant asphalt concrete. Furthermore, the rutting resistance of the asphalt concrete was strongly enhanced with increasing polyolefin dosage. However, a key challenge arises from the inherent crystallinity of polyolefins and their divergent molecular structure and weight compared to asphalt, which often leads to poor compatibility. This compatibility issue is particularly problematic in the dry method, which provides insufficient shear to disperse polyolefins effectively. Since dispersibility is crucial for the comprehensive performance of modified asphalt mixtures. Xia Lei et al. [22] successfully utilized waste polyolefin materials, including discarded mulch film, as a substitute for traditional SBS modifiers. The results demonstrated that the polyolefin-modified asphalt exhibited more than a two-fold improvement in high-temperature rutting resistance, alongside enhancements exceeding 30% in both low-temperature cracking resistance and aging resistance. Beyond homopolymers, olefin copolymers have also shown considerable promise as asphalt modifiers. For example, Zhu et al. [23] recently investigated a styrene–butyl acrylate (ST-BA) random copolymer modified asphalt, reporting that a 2 wt% copolymer content increased the ductility at 10 °C from 16 cm to 136 cm. At a 6 wt% content, the creep recovery rate increased by 12.1%, while the creep stiffness decreased by 23.5%, indicating significant improved low-temperature crack resistance. Collectively, such research provides a valuable foundation for developing high-performance polyolefin modifiers. An ideal modifier would significantly enhance the performance while maintaining the cost-effectiveness. Therefore, utilizing discarded polyolefin plastics to prepare modifiers presents a strategic approach that addresses three key challenges: (1) balancing high- and low-temperature performance, (2) improving dispersibility within the asphalt matrix, and (3) optimizing overall cost-effectiveness. This approach concurrently supports resource recycling and sustainable development. These three challenges form the core research objectives of the present study.

This study proposes a novel polyolefin composite modifier designed to simultaneously address multiple challenges: high-temperature rutting resistance, low-temperature crack resistance, dispersibility, and cost-effectiveness. Its design leverages the complementary properties of plastics (high hardness) and rubber (high toughness). Specifically, recycled plastics (e.g., EVA, ABS) were compounded with waste tire crumb rubber. A novel polyolefin composite modifier (PCM-H) was subsequently prepared by incorporating additives such as petroleum resin, polymers, and a crosslinking agent to induce a crosslinked copolymer structure. For comparative analysis, two commercially available polyolefin composite modifiers (PCM-1 and PCM-2) were selected. The chemical composition, thermal stability, and compatibility of these modifiers were investigated using Fourier transform infrared spectroscopy (FTIR) and differential scanning calorimetry (DSC). The optimal dosage of the PCM was determined. Various polyolefin composite modified asphalts were prepared by incorporating the polyolefin composite modifiers into an SBS modified asphalt base. The conventional, viscoelastic, aging, and rheological properties of those composite binders were systematically evaluated and compared. Microscopic morphology and chemical characteristics were characterized using fluorescence microscopy and infrared spectroscopy, respectively. This research aims to provide an effective solution for mitigating premature pavement distress under high-temperature and heavy-load conditions, thereby contributing to the sustainable development of transportation infrastructure.

## 2. Materials and Methods

### 2.1. Materials

The primary materials used to prepare the polyolefin composite modified asphalt in this study were 90 # base asphalt (supplied by Jingbo Petrochemical Co., Ltd. Zibo, China), SBS 501 modifier (supplied by LG Chem Ltd. Seoul, Korea), and PCM. The SBS modified asphalt (SBS MA) was prepared in the laboratory. Its composition was 94 wt% 90 # base asphalt, 4 wt% SBS 501, 2 wt% rubber oil, and 0.15 wt% sulfur-based stabilizer, with all percentages based on the total blend mass. The fundamental properties of the laboratory-prepared SBS MA are summarized in Table 1.

PCM-H was developed in the laboratory, and it presented as light-yellow particles. Its primary chemical constituents are detailed in Table 2. For comparison, two commercial polyolefin composite modifiers, PCM-1 and PCM-2, were procured from the China Highway (Beijing) Engineering Materials Technology Co., Ltd. Beijing, China. A comparative summary of their fundamental properties is presented in Table 3.

**Figure 1:**
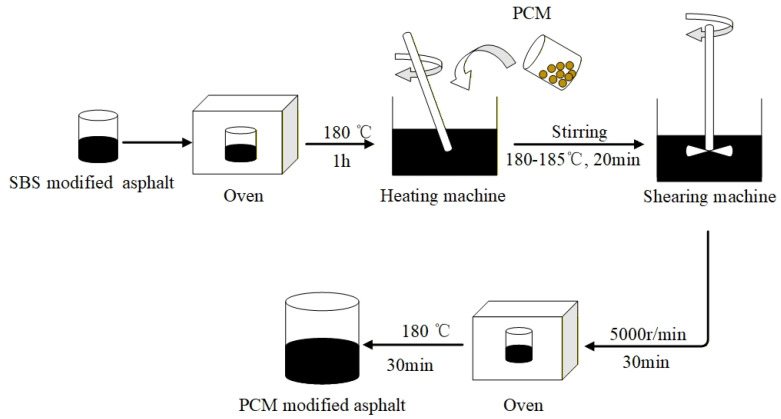
Preparation process of polyolefin composite modified asphalt.

The polyolefin composite modified asphalt was prepared according to the following procedure. First, the SBS MA was heated until molten in an oven at 180 °C for 1 h. Subsequently, predetermined amounts of respective polyolefin composite modifiers were weighed. The SBS MA was then quickly transferred to a heating jacket maintained at 180–185 °C. Once the asphalt reached 180 °C, the polyolefin composite modifier was gradually incorporated and manually stirred with a glass rod for 20 min. The mixture was then sheared using a high-shear mixer at 5000 rpm for 30 min to ensure homogeneous dispersion. Finally, the blend was placed in an oven at 180 °C for 30 min to produce the final polyolefin composite modified asphalt. A schematic of the preparation process is presented in Figure 1.

### 2.2. Test Methods

#### 2.2.1. Conventional Performance Test of Composite Modified Asphalt

The conventional performance of the polyolefin composite modified asphalt was comprehensively evaluated following the standard test methods specified in the Standard Test Methods of Asphalt and Asphalt Mixture for Highway Engineering (JTG 3410-2025), [24].

#### 2.2.2. Microscopic Analysis of Polyolefin Composite Modifier

(1) FTIR Test

The FTIR test was performed using a Nicolet 6700 FTIR spectrometer (Thermo Fisher Scientific, Waltham, MA, USA). Approximately 3 mg of each polyolefin composite modifier was finely ground and homogenized with potassium bromide (KBr) and then compressed into a translucent pellet for analysis. The spectral resolution was set to 4 cm^−1^, with 32 scans accumulated over a wavenumber range of 400 to 4000 cm^−1^.

(2) Thermogravimetric Test

TGA was conducted using a TGA 2 thermal analyzer (Thermo Fisher Scientific, Waltham, MA, USA). Tests were performed under a protective atmosphere with a flow rate of 50 mL/min. The temperature was programmed to increase from ambient temperature to 800 °C at a constant heating rate of 10 °C/min.

(3) Differential Scanning Calorimetry (DSC) Test

DSC analysis of the modifiers was conducted using a DSC Q200 instrument (TA Instruments, New Castle, DE, USA) under a nitrogen atmosphere at a flow rate of 50 mL/min. Measurements were performed over a temperature range from −30 °C to 300 °C, at a constant heating rate of 10 °C/min.

#### 2.2.3. Fluorescence Microscopic Dispersion Observation Test

The microscopic morphology of the polyolefin composite modified asphalt was observed using a Zeiss Stemi 508 stereomicroscope (Carl Zeiss AG, Oberkochen, Germany). A small aliquot of the asphalt sample was placed on a glass slide, promptly covered with a coverslip, and heated on a hot plate at approximately 150 °C. It was then compressed into a thin translucent film using a manual press. The prepared specimen was then examined under the microscope to observe the dispersion behavior of the polyolefin composite modifier.

#### 2.2.4. Gel Permeation Chromatography (GPC) Test

The molecular weight distribution of the composite modified asphalt was analyzed by GPC using an LC20AD system (Shimadzu Corporation, Kyoto City, Japan). Tetrahydrofuran (THF) (HPLC-grade, TEDIA, Fairfield, OH, USA) was used as the eluent at a flow rate of 1.0 mL/min, with the column temperature maintained at 35 °C.

#### 2.2.5. Rheological Performance Test of Composite Modified Asphalt

The dynamic rheological properties of the different asphalt samples were evaluated using a dynamic shear rheometer (DSR, Anton Paar, Sydney, Austria). A comprehensive test series was performed, comprising high-temperature performance grading (PG), bending beam rheometer (BBR) test, temperature sweep, a multiple stress creep recovery (MSCR) test, and a time sweep test.

(1) High-temperature performance grading (PG) test

Approximately 1.0 g of the asphalt binder was uniformly loaded between 25 mm diameter parallel plates with a 1 mm gap. Testing was performed at a fixed angular frequency of 10 rad/s. The test commenced at a starting temperature of 58 °C, with measurements of the complex shear modulus (G*), phase angle (δ), and rutting factor (G*/sin δ) recorded at 6 °C intervals. Three replicate specimens were tested for each condition. The high-temperature grade was determined according to the criterion requiring a rutting factor (G*/sinδ) of the original asphalt to be ≥1.0 kPa and that of the RTFOT residue to be ≥2.2 kPa [25].

(2) Low-temperature bending creep stiffness test

The low-temperature properties of the polyolefin composite modified asphalt were characterized using a bending beam rheometer (BBR; Cannon Instrument Company, State College, PA, USA). Beam specimens (127 mm × 12.7 mm × 6.35 mm) were subjected to a constant load for 240 s at temperatures of −12 °C, −18 °C, and −24 °C. The creep stiffness (S) and creep rate (m-value) at a loading time of 60 s were used to determine the low-temperature grade, based on the criteria S ≤ 300 MPa and m ≥ 0.30.

(3) Temperature sweep test

Temperature sweep tests were performed at a fixed frequency of 10 rad/s and a stress of 100 Pa over a temperature range of 28 to 82 °C. Data were recorded at 6 °C intervals. Three replicate specimens were tested. The viscoelastic properties of the modified asphalt were characterized by the rutting factor (G*/sin δ) and phase angle (δ).

(4) Multiple Stress Creep Recovery (MSCR) Test

The MSCR test was conducted on asphalt using a DSR. At a test temperature of 60 °C, two stress levels (0.1 kPa and 3.2 kPa) were applied. For each stress level, a creep load was applied for 1 s followed by a 9 s recovery period. This cycle was repeated 10 times. Two replicate tests were performed per sample [26].

#### 2.2.6. Self-Healing Test

Time sweep tests were performed on different asphalt samples using a “damage–healing–damage” model to simulate the self-healing process. Three damage levels (D1, D2, and D3) were defined, corresponding to reductions in the norm of the complex shear modulus (|G*|) by 15%, 20%, and 25%, respectively. A healing interval of 300 s was implemented to facilitate the self-healing of the asphalt. The healing index, A′, for each damage level was calculated using Equation (1). A higher A′ value indicated a higher healing capacity and enhanced fatigue resistance of the asphalt.

(1) $$A^{’}=\frac{G_{2}-G_{1}}{G_{0}-G_{1}}\times \frac{t_{1}}{t_{2}},$$

where G_0_ is the initial complex shear modulus (kPa); G_1_ is the complex shear modulus at the end of the first damage phase (kPa); G_2_ is the complex shear modulus at the beginning of the second damage phase after the 300 s healing period (kPa); t_1_ is the duration for the modulus to decrease from G_0_ to G_1_ during the first damage phase (s). t_2_ is the duration for the modulus to decrease from G_2_ to G_1_ during the second damage phase (s).

## 3. Results and Discussion

### 3.1. Composition and Microscopic Dispersion Behavior of PCM-H

#### 3.1.1. Composition of Polyolefin Composite Modifier

**Figure 2:**
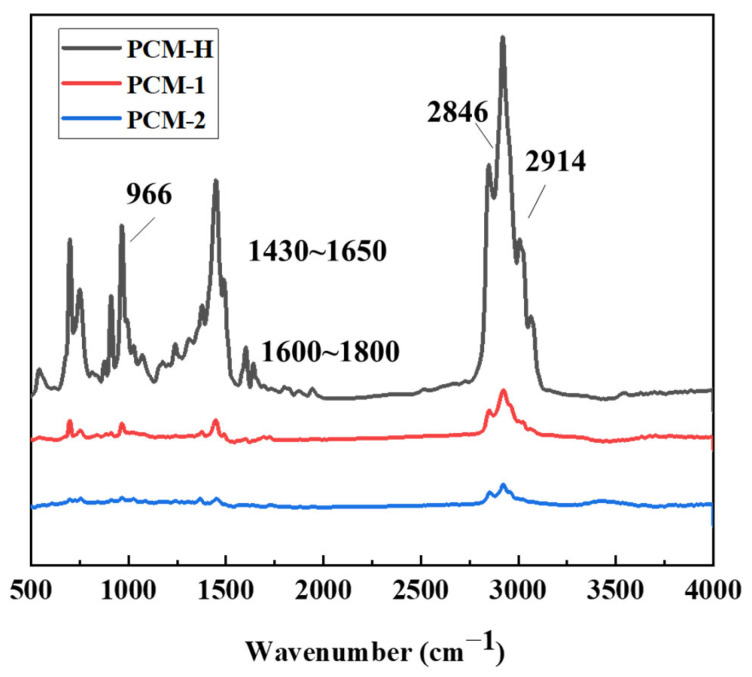
Infrared spectra of PCM-H and two commercially available polyolefin composite modifiers (PCM-1 and PCM-2).

FTIR testing was conducted on the novel polyolefin composite modifier (PCM-H) and the two commercial modifiers (PCM-1 and PCM-2) for comparative analysis. The resulting spectra are presented in Figure 2.

As shown in Figure 2, all three polyolefin composite modifiers exhibit distinct asymmetric stretching vibration peaks for -CH_2_ and C-H at approximately 2914 cm^−1^ and 2846 cm^−1^. PCM-H shows the most pronounced peaks in this region, indicative of a higher concentration of aliphatic hydrocarbon chains. The absorption peaks in the 1430–1650 cm^−1^ region are assigned to C=C stretching vibrations from aromatic rings. Peaks in the 1600–1800 cm^−1^ region correspond to carbonyl (C=O) stretching vibrations. The peak at 966 cm^−1^ is characteristic of the twisting vibration of trans C=C bonds, while the peak at 911 cm^−1^ corresponds to the out-of-plane rocking vibration of =CH_2_ groups. Peaks at 699 cm^−1^ and 757 cm^−1^ are attributed to vibrations of monosubstituted benzene rings. PCM-H exhibits particularly prominent absorption peaks at these characteristic positions, suggesting a higher relative content of styrene–butadiene structures within its polymer composition. This is consistent with the formulation of PCM-H, which incorporates significant proportions of waste tire rubber powder, petroleum resin, and SBS-based polymers. These raw materials undergo intensive shear mixing and thermal processing during high-temperature melt extrusion. This process can lead to the breakdown of some macromolecular substances, potentially increasing the content of branched alkane components. The absorption peak positions of the modifier PCM-1 are generally consistent with that of PCM-H but show weaker intensity in the fingerprint region and at the benzene ring absorption peaks. This suggests a lower relative content of SBS and other aromatic polymers. In contrast, PCM-2 shows markedly weaker absorption at 966 cm^−1^, indicating a relatively lower polyolefin content.

#### 3.1.2. Thermal Stability and Compatibility of Polyolefin Composite Modifiers

**Figure 3:**
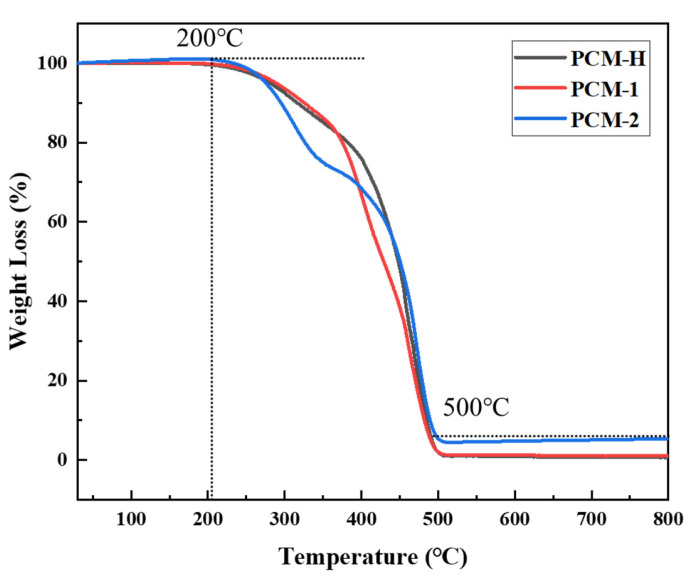
Comparison of thermogravimetric behavior of PCM-H and two commercially available polyolefin composite modifiers (PCM-1 and PCM-2).

The thermal degradation behavior of the three polyolefin composite modifiers was investigated using a thermogravimetric analyzer (TGA). The resulting thermogravimetric (TG) curves are compared in Figure 3.

As shown in Figure 3, the pyrolysis process for all three modifiers occurs in three distinct stages. The first stage, below 200 °C, is attributed primarily to the loss of absorbed moisture. During this stage, the mass loss rate is minimal, and the TG curves are relatively stable, consistent with the absence of hydrated compounds in the modifiers. The second stage (200–500 °C) represents the primary pyrolysis interval, characterized by a sharp decline in TG curves, indicating rapid mass loss. The rapid mass loss is attributed primarily to the thermal cleavage of polymer chains and secondary volatilization reactions. Within this interval, PCM-2 exhibits the most rapid decomposition onset at a lower temperature, whereas PCM-H demonstrates the most gradual initial mass loss profile. This observation is consistent with the FTIR analysis, which suggested that the high-temperature processing of PCM-H leads to chain scission, generating lighter volatile components such as alkanes. Consequently, the mass loss of PCM-H in this stage is dominated by the volatilization of these pre-formed light components. In contrast, PCM-2 undergoes simultaneous chain scission and volatilization, resulting in a more abrupt mass loss. This thermal behavior further indicates that PCM-H possesses a more stable polyolefin-rich composition from a thermodynamic standpoint.

**Figure 4:**
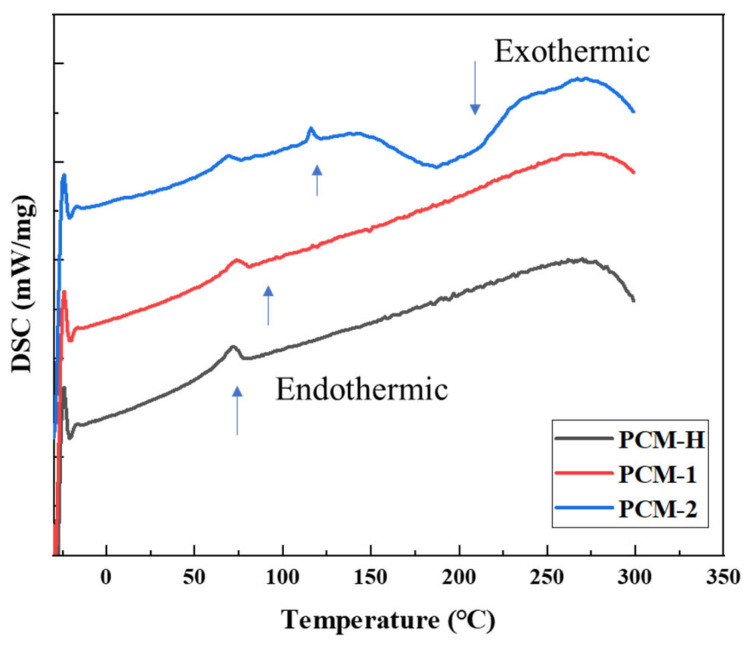
Thermal stability analysis of different polyolefin composite modifiers.

The thermal behavior and compatibility of the polyolefin composite modifiers were further analyzed using differential scanning calorimetry (DSC). DSC analysis was performed on PCM-H, PCM-1, and PCM-2, with the results are presented in Figure 4.

As shown in Figure 4, all three polyolefin modifiers exhibit endothermic transitions below 150 °C. Since asphalt modification is typically conducted at 180–185 °C, the addition of these modifiers lowers the initial temperature, indicating that the modifiers undergo phase changes (e.g., melting, glass transition), facilitating their integration into the asphalt system. At higher temperature, only PCM-2 exhibits a distinct exothermic peak, observed between 180 and 200 °C. This exotherm suggests a recrystallization event, which can induce phase separation and compromise the stability of the modified asphalt system. In contrast, PCM-H and PCM-1 show no significant thermal events in the 150–200 °C range. This absence of exotherms indicates a lack of detrimental recrystallization. This thermal behavior suggests better compatibility with the asphalt matrix and contributes to higher system stability during high-temperature processing.

### 3.2. Research on the Optimal Dosage of PCM-H

**Figure 5:**
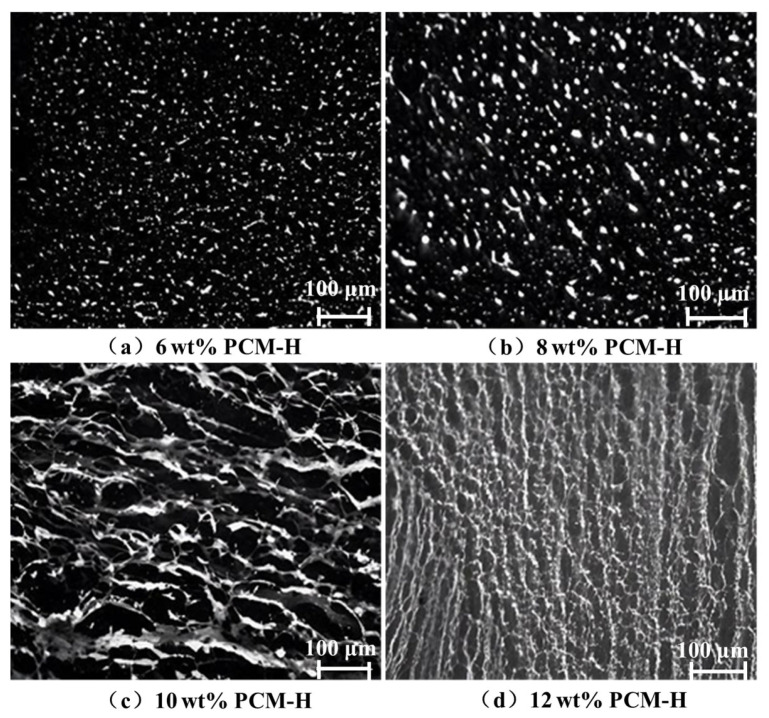
Microscopic dispersion behavior of different dosages of PCM-H. (**a**) 6 wt% PCM-H; (**b**) 8 wt% PCM-H; (**c**) 10 wt% PCM-H; (**d**) 12 wt% PCM-H.

Polyolefin composite modified asphalt samples were prepared by incorporating PCM-H into an SBS MA base at dosages of 6, 8, 10, and 12 wt% via high-shear blending. The dispersion state and compatibility of the modifier within the asphalt were examined using fluorescence microscopy; representative micrographs are presented in Figure 5.

Figure 5 shows that at PCM-H contents below 8 wt%, diffusion and adsorption between the SBS MA and PCM-H are incomplete, inhibiting the formation of a continuous polymer network. In this regime, the asphalt constitutes the continuous phase, while PCM-H remains as the dispersed phase. At a PCM-H content of 10 wt%, the modifier absorbs the maltene fractions from the SBS MA, leading to full swelling and uniform dispersion. This facilitates the development of an interconnected crosslinked network structure. Consequently, the system transitions to a co-continuous morphology, where PCM-H and the SBS MA form homogeneous interpenetrating networks. However, at 12 wt% PCM-H, a phase inversion occurs: the SBS MA becomes the dispersed phase, encapsulated within the continuous swollen PCM-H network. This excessive modifier content significantly increases the system viscosity, impairing the shear processing and leading to inhomogeneous dispersion and agglomeration. Therefore, a PCM-H dosage of 10 wt% is identified as optimal for achieving a well-dispersed co-continuous morphology within the SBS MA.

### 3.3. Microscopic Dispersion Behavior of PCM-H in Asphalt

**Figure 6:**
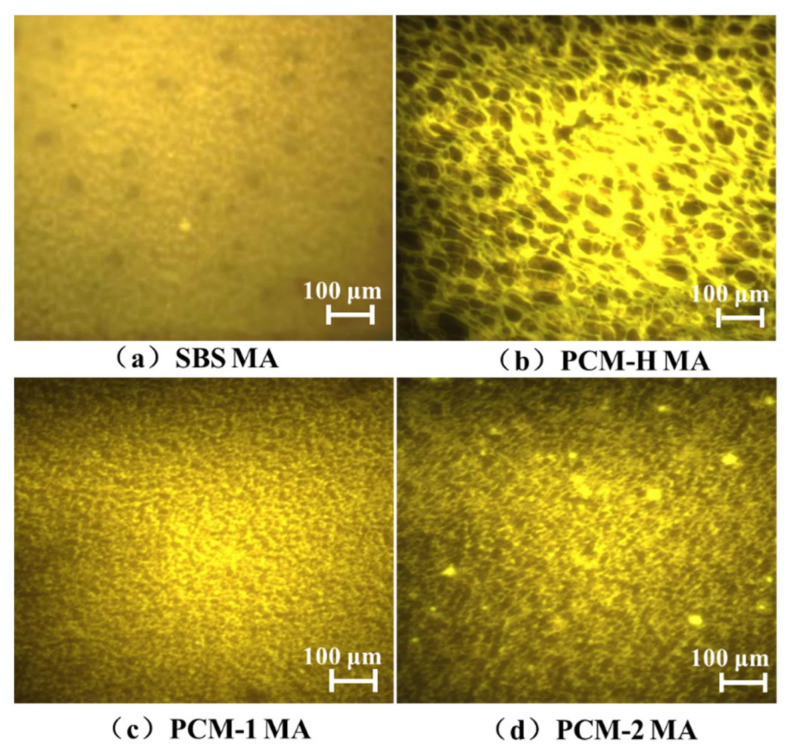
Microscopic distribution morphology of three different polyolefin composite modified asphalts. (**a**) SBS MA; (**b**) PCM-H MA; (**c**) PCM-1 MA; (**d**) PCM-2 MA.

Using SBS MA as the base binder, three polyolefin composite modifiers (PCM-H, PCM-1, and PCM-2) were each added at a dosage of 10 wt% to prepare three distinct modified asphalts, designated PCM-H MA, PCM-1 MA, and PCM-2 MA. The morphological distribution of the SBS MA and the three composite modified asphalts was examined using fluorescence microscopy, as shown in Figure 6.

As shown in Figure 6, the SBS MA exhibits uniformly dispersed modifiers but fails to form a distinct crosslinked network structure. In the PCM-H MA, the modifier undergoes shear-induced swelling and adsorption, crosslinking with the SBS MA to form a pronounced interpenetrating network with a textured morphology. Meanwhile, PCM-1 exhibits a bicontinuous phase structure, indicating mutual penetration and integration between the modifier and the SBS MA. In the PCM-2 MA, the modifier is dispersed but forms a network with weak interconnections. Evidence of partial agglomeration, incomplete dissolution, and crystalline precipitation is observed, indicating distinct phase interfaces and poor compatibility. This morphological observation is consistent with the DSC results, which indicated a recrystallization event for PCM-2 between 180 and 200 °C, a process detrimental to system stability. Under high-temperature shearing at 180 °C, PCM-H more effectively deals with the maltene fractions of asphalt, leading to swelling and dissolution. Facilitated by stabilizers, it develops a robust crosslinked interpenetrating network. This structure signifies excellent compatibility with the asphalt binder, which is the fundamental mechanism for the enhanced overall performance of PCM-H MA.

### 3.4. Relative Molecular Weight Distribution of PCM-H MA

GPC tests were performed on SBS MA, PCM-1 MA, PCM-2 MA, and PCM-H MA. The relative molecular weight distributions are summarized in Table 4.

According to Table 4, the addition of polyolefin composite modifiers into SBS MA increased the overall molecular weight. While the number-average molecular weight (Mₙ) showed minimal change, the z-average (M_2_) and z+1-average (M_2_₊_1_) molecular weights increased substantially. This trend indicates that during the high-shear blending process, the polyolefin composite modifiers melted, dispersed, and subsequently re-formed into a tightly crosslinked network structure again with the SBS MA. Among them, PCM-H MA exhibited the highest polydispersity index (PDI). This suggests a higher population of lower molecular weight substances within its structure, aligning with the FTIR analysis of an increased content of branched alkane components. Similarly, the relatively high PDI of PCM-2 MA is consistent with the thermal analysis (DSC) result, which showed that PCM-2 is prone to crystallization, a process that can generate a broader distribution of molecular weights.

### 3.5. Comparative Study on Conventional Properties of Polyolefin Composite Modified Asphalt

The basic properties of different polyolefin composite modified asphalts were tested and compared with those of the SBS MA, as summarized in Table 5.

As shown in Table 5, the incorporation of polyolefin composite modifiers significantly improves both the high- and low-temperature performance of SBS MA. Among them, PCM-H MA demonstrates the most remarkable enhancement. Its dynamic viscosity at 60 °C reaches approximately 2,846,151 Pa·s, which represents an increase by a factor of 111 compared to the conventional SBS MA and far exceeds the technical requirements for high viscosity asphalt. Following RTFOT, it maintains a ductility higher than 30 cm at 5 °C. PCM-1 MA ranks second in performance improvement, substantially enhancing both high- and low-temperature properties and achieving a dynamic viscosity of approximately 2,151,612 Pa·s at 60 °C. The powdered PCM-2 modifier increases the softening point of SBS MA to 95.6 °C and improves the 5 °C ductility to 34 cm. However, it results in a penetration below 40 mm and provides only a limited enhancement in 60 °C dynamic viscosity. This can be attributed to the tendency of PCM-2 to crystallize and precipitate, creating phase interfaces that compromise system compatibility, as discussed in the thermal and morphological analyses. The Brookfield viscosity at 175 °C for all modified asphalts meets specification requirements, with PCM-H MA exhibiting the highest value and PCM-2 MA the lowest. This phenomenon is due to the composition of PCM-H, which contains thermoplastic rubber–plastic elastomers that readily melt and disperse under high-temperature shear, subsequently undergoing recrosslinking to form an interpenetrating network. This network enhances internal cohesion and aging resistance. In contrast, the powdered PCM-2 has a high specific surface area and tends to partially crystallize within the SBS MA, creating phase interfaces and resulting in a weakly connected crosslinked network structure with uneven stress distribution, ultimately leading to localized hardening.

### 3.6. Study on Rheological Properties of Polyolefin Composite Modified Asphalt

#### 3.6.1. High Temperature Grading Performance of Polyolefin Composite Modified Asphalt

**Figure 7:**
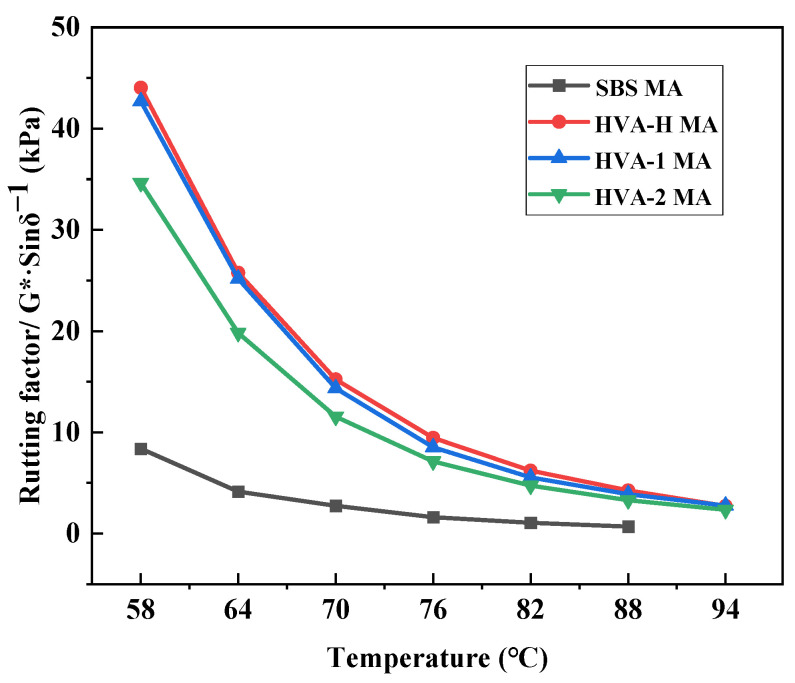
The changes in G*/(sin δ) of SBS MA and different polyolefin composite modified asphalts with temperature.

Asphalt is a temperature-sensitive viscoelastic material whose properties vary significantly with temperature. The phase angle (δ) quantifies the relative proportion of viscous (non-recoverable) deformation, whereas the complex shear modulus (G*) r characterizes the material’s total resistance to deformation under repeated shear stress. The rutting factor (G*/sin δ) indicates the level of rutting resistance, where higher values correspond to superior resistance to permanent deformation at high service temperatures. The variation in G*/sin δ with temperature for the various polyolefin composite modified asphalts is illustrated in Figure 7.

As shown in Figure 7, the rutting factor (G*/sin δ) for all samples decreases with increasing temperature. All three composite modified asphalts exhibit significantly higher G*/sin δ values compared to SBS MA. Among them, PCM-H MA demonstrates the highest G*/sin δ value, followed by PCM-1 MA, whereas PCM-2 MA shows the lowest values, consistent with the performance trends observed in conventional tests.

As shown in Table 6, high-temperature performance grading (PG) tests were conducted on both original asphalts and the TFOT residual asphalt, and the results are summarized.

As shown in Table 6, the high-temperature grade of SBS MA is 76 °C. Following modification with the polyolefin composite modifiers, all three modified asphalts exhibit a significant improvement in their high-temperature grades. Among them, the high-temperature grade increased to 88 °C for PCM-2 MA, and 94 °C for both PCM-H MA and PCM-1 MA. Furthermore, the G*/sin δ for PCM-H MA was higher than that of PCM-1 MA at equivalent temperatures, both before and after TFOT. This result indicates that PCM-H is more effective in enhancing the high-temperature performance of asphalt binder. This superior performance is attributed to the crosslinked interpenetrating network structure formed by PCM-H within the SBS MA, as revealed by the microstructural analysis.

#### 3.6.2. Analysis of Temperature Scanning Test

**Figure 8:**
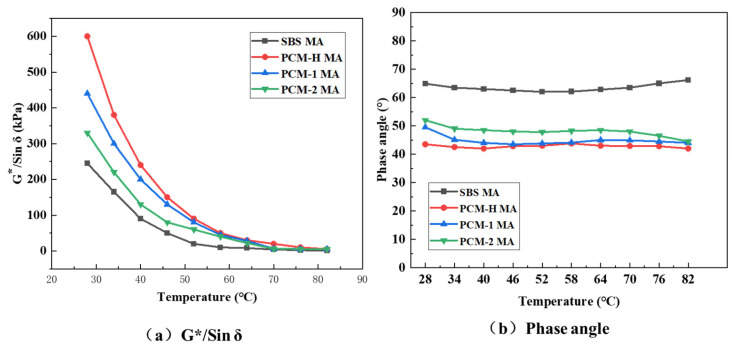
The changes in G*/Sin δ and δ of different asphalts with temperature. (**a**) G*/Sin δ; (**b**) δ.

To investigate the modification mechanism and phase structure, the wide-range temperature–modulus curves were employed for the different polyolefin composite modified asphalts. The temperature sweep test results for SBS MA and different polyolefin composite modified asphalts are presented in Figure 8.

As shown in Figure 8a, at temperature below 64 °C, the complex shear modulus of both SBS MA and the polyolefin composite modified asphalts decrease rapidly with increasing temperature, indicating a transition from a viscoelastic solid to a viscous fluid state. When the temperature exceeds 64 °C, the phase angle remains below 90 °C with an insignificant increase, suggesting that these asphalts still exhibit properties of viscous fluids at high temperatures while maintaining excellent elastic recovery performance. With further temperature increase, the G*/sin δ of PCM-2 MA and PCM-1 MA converges with that of SBS MA, whereas the value for PCM-H MA remains significantly higher than all other samples. Notably, the modulus–temperature sweep curve for PCM-H MA exhibits a phenomenon where the modulus remains constant with increasing temperature. Generally, the phase angle increases with the rising temperature, indicating enhanced plastic behavior. However, except for SBS MA, the phase angles of the polyolefin composite modified asphalts decrease rather than increase at high temperatures. Among them, PCM-H MA and PCM-1 MA exhibit a more pronounced decrease in phase angle, while PCM-2 MA shows a relatively stable phase angle. This indicates that the polyolefin composite modified asphalts maintain a favorable balance of elasticity and viscosity at high temperatures. This rheological characteristic is a key factor in the superior and balanced high- and low-temperature performance of PCM-H MA. These temperature sweep test results align with the conventional performance analysis of the modified asphalts mentioned earlier.

#### 3.6.3. Analysis of Low-Temperature Crack Resistance

**Figure 9:**
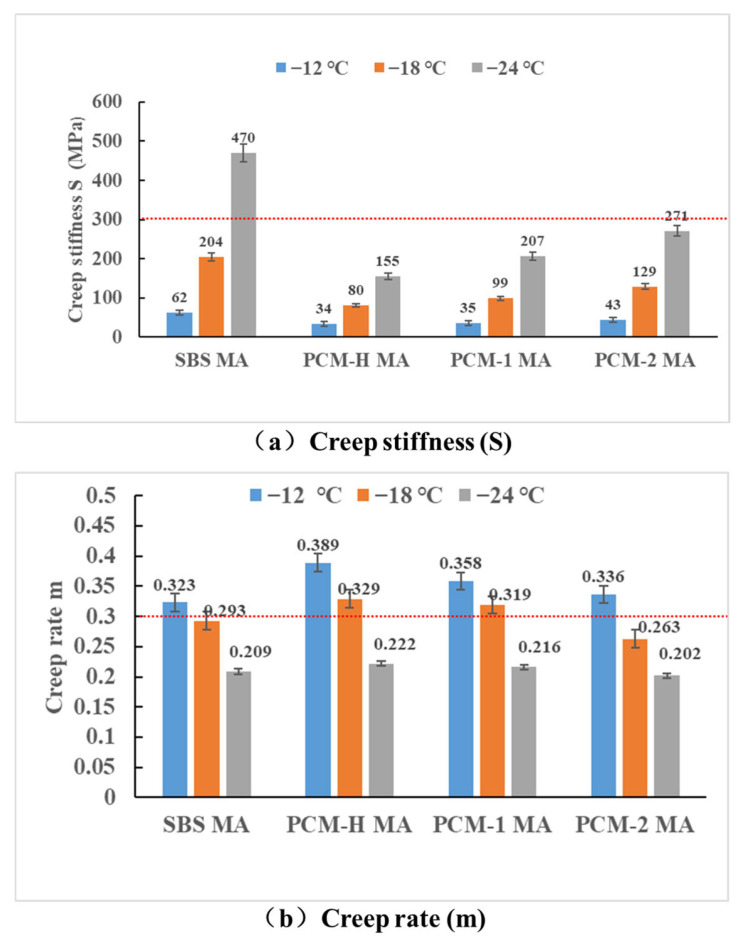
Results of BBR test of different polyolefin composite modified asphalts. (**a**) Creep stiffness (S); (**b**) creep rate (m).

Research from the Strategic Highway Research Program (SHRP) has demonstrated that the low-temperature creep stiffness (S) and creep rate (m-value) derived from the Bending Beam Rheometer (BBR) test are closely correlated with the low-temperature fracture temperature of asphalt mixtures. The BBR test was used to evaluate the low-temperature crack resistance of different asphalt binders. Figure 9 presents the S-values and m-values for SBS MA and the polyolefin composite modified asphalts at test temperatures of −12 °C, −18 °C, and −24 °C.

As shown in Figure 9, lower test temperatures result in a higher creep stiffness (S-value) and a lower m-value. This trend aligns with the fundamental rheological principles of using the stiffness modulus and creep rate to characterize the rheological and stress relaxation properties of asphalt materials under low-temperature conditions and constant load pressure. Based on the specification criteria (S ≤ 300 MPa and m ≥ 0.3), both SBS MA and PCM-2 MA meet the low-temperature grade of −12 °C, while PCM-H MA and PCM-1 MA surpass the low-temperature grade of −18 °C. The materials can be ranked by increasing stiffness as PCM-H MA < PCM-1 MA < PCM-2 MA < SBS MA. Conversely, the ranking for the creep rate is PCM-H MA > PCM-1 MA > SBS MA ≈ PCM-2 MA. These results demonstrate that the polyolefin composite modified asphalts, particularly PCM-H MA and PCM-1 MA, exhibit significantly improved stress relaxation performance and low-temperature flexibility compared to SBS MA. In contrast, PCM-2 MA exhibits low-temperature crack resistance comparable only to that of SBS MA. The enhanced performance of PCM-H MA and PCM-1 MA is attributed to their high compatibility with the asphalt, which effectively reduces the stiffness and increases the flexibility, thereby achieving optimal low-temperature crack resistance. Combined with the aforementioned microscopic analysis, the reduced compatibility of PCM-2 MA due to partial crystallization and precipitation in the asphalt system adversely affects its low-temperature crack resistance.

#### 3.6.4. Analysis of Permanent Deformation Resistance of Polyolefin Composite Modified Asphalt

**Figure 10:**
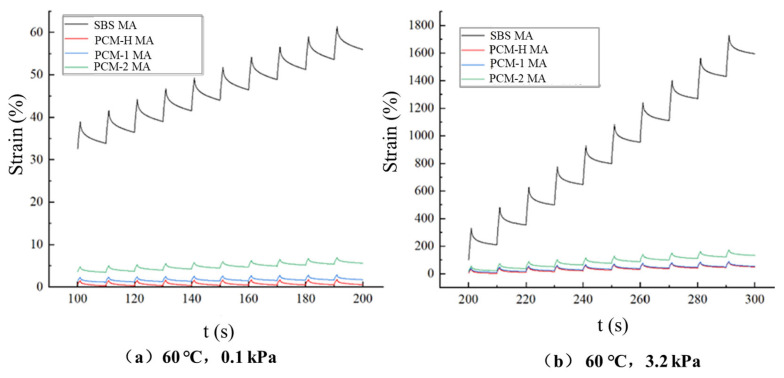
The cumulative creep strain of SBS MA and polyolefin composite modified asphalt with the change in load time. (**a**) 60 °C, 0.1 kPa; (**b**) 60 °C, 3.2 kPa.

The non-recoverable creep compliance (Jnr) and creep recovery rate (R) of polyolefin composite modified asphalt were evaluated in the MSCR test at 60 °C, and the cumulative creep strains (γ) of SBS MA and polyolefin composite modified asphalts under different stress levels are illustrated in Figure 10.

As shown in Figure 10, at the test temperature of 60 °C and stress levels of 100 Pa and 3200 Pa, both the peak and residual strains of the asphalts gradually increase with the increasing number of loading cycles. For both the peak strain per cycle and the residual strain after 10 cycles, the asphalts can be ranked as follows: SBS MA > PCM-2 MA > PCM-1 MA > PCM-H MA. Notably, at the higher stress level (3.2 kPa), the residual strain of PCM-1 MA approaches that of PCM-H MA. However, PCM-H MA consistently exhibits the lowest residual strain, indicating its superior resistance to permanent deformation and enhanced elastic recovery. This superior performance is attributed to the behavior of PCM-H during high-temperature blending. At 180 °C, PCM-H effectively absorbs the maltene fractions, leading to swelling and dissolution. Facilitated by chemical stabilizers, this process promotes the formation of a stable crosslinked interpenetrating network within the asphalt.

The MSCR test utilizes two parameters to evaluate the deformation resistance of asphalt: the recovery rate (R) and the non-recoverable creep compliance (Jnr), defined in Equations (2) and (3). The recovery rate (R) represents the asphalt’s elastic recovery following the removal of stress, thereby highlighting its elastic characteristics. In contrast, Jnr provides a more direct and performance-relevant measure of rutting resistance than the conventional rutting factor (G*/sin δ).

(2) $$R_{\tau }=\frac{r_{pi}-r_{nri}}{r_{pi}-r_{oi}},$$

(3) $$J_{nr\tau i}=\frac{r_{nri}}{\tau },$$

where r_pi_ represents the peak strain, r_nri_ is the residual strain, r_0i_ denotes the initial strain in each loading cycle, and τ indicates the creep stress.

**Figure 11:**
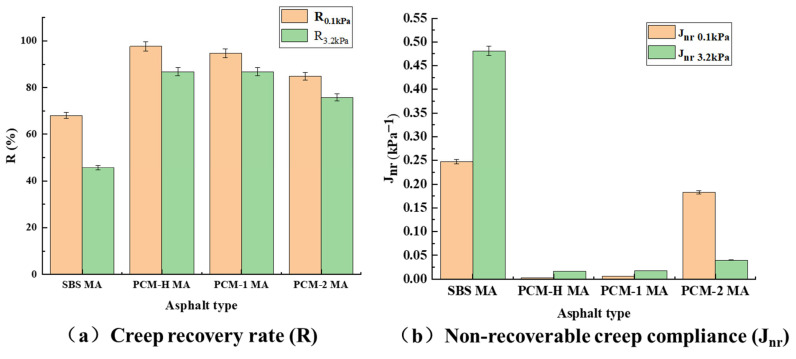
Creep recovery rate and non-recoverable creep compliance of modified asphalt under different stress levels. (**a**) Creep recovery rate (R); (**b**) non-recoverable creep compliance (J_nr_).

The average R and Jnr values at each stress level were calculated according to Equations (4) and (5). The results of these calculations are presented in Figure 11.

(4) $$R_{\tau }=\frac{\sum_{i=1}^{10}R_{\tau i}}{10}$$

(5) $$J_{nr\tau }=\frac{\sum_{i}^{10}R_{\tau i}}{10}$$

As shown in Figure 11a, the modified asphalt binders exhibit distinct creep recovery capabilities at both stress levels. The recovery capability can be ranked as: PCM-H MA ≈ PCM-1 MA > PCM-2 MA > SBS MA. It demonstrates that the polyolefin composite modifiers significantly enhance the elastic recovery capability of SBS MA. The non-recoverable creep compliance (Jnr) can more accurately characterize the high-temperature rutting resistance of asphalt. A higher Jnr value indicates higher accumulated permanent deformation and, consequently, weaker high-temperature rutting resistance. As shown in Figure 11b, the Jnr values at 0.1 kPa and 3.2 kPa are ranked as PCM-H MA < PCM-1 MA < PCM-2 MA < SBS MA. This indicates that PCM-H MA possesses the strongest high-temperature rutting resistance among all samples. These MSCR results are consistent with the performance trends identified through the conventional rutting factor (G*/sin δ) and low-temperature grading. This consistency confirms that PCM-H simultaneously and substantially enhances both the high-temperature rutting resistance and the low-temperature crack resistance of the asphalt binder.

#### 3.6.5. Study on Self-Healing Capacity

**Figure 12:**
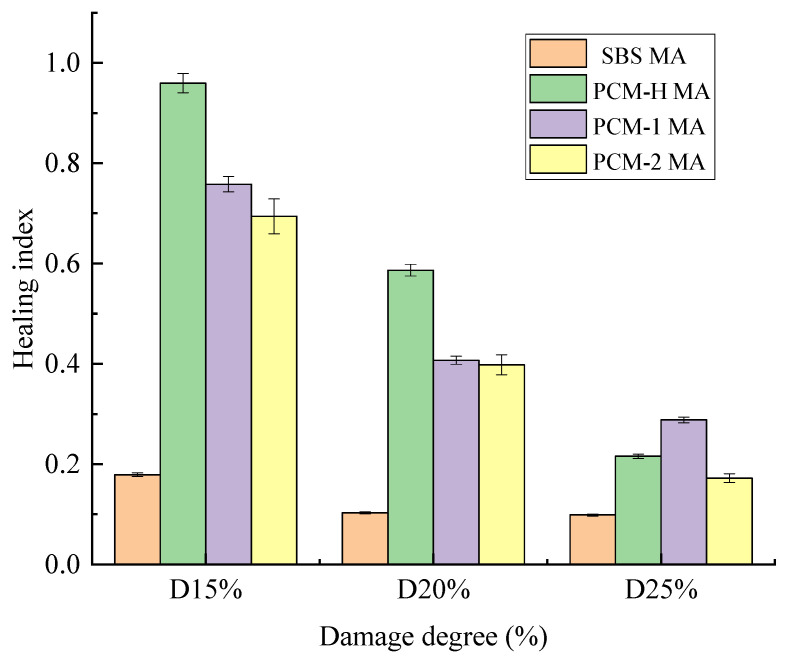
Healing index (A′) corresponding to the same degree of injury.

Fatigue cracking, which initiates primarily within the asphalt binder, poses a critical challenge in pavement engineering due to its detrimental impact on the service life and structural integrity. Owing to its viscoelastic rheological nature, asphalt possesses a self-healing capability when external loads are removed. This allows for the partial recovery of mechanical strength, thereby extending the fatigue life of asphalt pavements. The phenomenological parameter, the healing index (A′), serves as a reliable metric for quantifying the healing capacity of asphalt materials. The healing index (A′) corresponding to different damage levels is presented in Figure 12, when a higher A’ value indicates a higher healing capacity and, consequently, superior fatigue resistance.

As observed in Figure 12, the healing index (A′) gradually decreases with increasing damage degree for a constant rest period, indicating that the self-healing performance of asphalt is significantly impaired by the extent of prior damage. More severe fatigue damage presents a stronger challenge to the healing process, a trend directly linked to the inherent deformation recovery resistance of each asphalt binder. For damage levels below 25%, the healing indices at equivalent damage are ranked as PCM-H MA > PCM-1 MA > PCM-2 MA > SBS MA. This demonstrates that PCM-H MA exhibits the most robust healing capacity and, consequently, the best fatigue resistance, within this damage range. However, at the highest tested damage level (25%), the healing index (A′) of PCM-1 MA surpasses that of PCM-H MA. This trend aligns with the earlier MSCR test results, where PCM-1 MA demonstrated creep recovery comparable to that of PCM-H MA under high stress. This behavior can be explained by the microstructure of these composites. The sufficient and uniform dispersion of modifiers enables extensive bonding and crosslinking, forming a stable network structure that enhances the overall viscoelasticity.

## 4. Discussion

This study systematically developed a novel polyolefin composite modifier (PCM-H) from recycled materials and evaluated its performance in SBS MA relative to two commercial benchmarks. The results confirm that PCM-H, characterized by a high styrene–butadiene content and favorable molecular weight distribution, exhibits superior compatibility and thermal stability. It forms a crosslinked interpenetrating three-dimensional network within the SBS MA. This structure is responsible for the observed superior performance, including enhanced high- and low-temperature performance, elastic response, and fatigue healing capability. The primary conclusions are as follows:

(1) Modifier Properties Determine the Compatibility: The efficacy of a polyolefin modifier is critically governed by its thermal stability and compatibility within the SBS MA. PCM-H exhibits stable behavior and good compatibility at standard modification temperatures. In contrast, PCM-2 shows a propensity to crystallize and precipitate within the 180–200 °C range, thereby destabilizing the modified system.
(2) Formation of a Reinforcing Network: The exceptional performance enhancement, particularly for PCM-H, is mechanistically attributed to the in situ formation of a crosslinked interpenetrating three-dimensional network structure within the asphalt. This network, developed through shear-induced swelling and subsequent crosslinking with SBS, significantly enhances the internal cohesion and mechanical integrity of the binder.
(3) Synergistic Performance Enhancement: The developed network structure facilitates a synergistic enhancement of asphalt properties. It provides substantial resistance to high-temperature deformation and rutting, evidenced by a dramatically increased dynamic viscosity and a high PG grade. Simultaneously, it maintains excellent low-temperature crack resistance, fatigue performance, and healing capability.
(4) Optimal Formulation and Performance: Comparative analysis confirms that asphalt modified with PCM-H at its optimal dosage of 10 wt% delivers the most balanced and superior overall performance. It sets a performance benchmark for high-temperature elasticity and fatigue-healing capacity. PCM-1 presents a competitive alternative, particularly under high-stress conditions where creep recovery is critical.

This study was conducted at a laboratory scale. The long-term field performance, including aging under real environmental conditions (e.g., UV radiation, moisture) and the impact on full-scale mixture properties, require further investigation.

## Figures and Tables

**Table 1:** Basic properties of laboratory-prepared SBS modified asphalt.

Properties		Unit	Technical Requirements	Value
penetration (25 °C, 100 g, 5 s)		0.1 mm	40–60	50.1
softening point		°C	≥60	80.7
ductility (5 cm/min, 5 °C)		cm	≥20	28.9
Brookfield viscosity (135 °C)		Pa·s	≤3	1.98
flash point		°C	≥230	245
solubility		%	≥99	99.5
after RTFOT	penetration ratio (25 °C)	%	≥65	81.9
	ductility (5 °C)	cm	≥15	16.8
	mass loss	%	≤±1.0	+0.08

**Table 2:** Composition of PCM-H polyolefin composite modifier developed in the laboratory.

Component	Content (wt%)
waste tire rubber	10
recycling EVA plastic	25
recycling ABS plastic	20
petroleum resin	15
SBS	20
naphthenic oil	10
crosslinking agents and other additives (added externally)	3

**Table 3:** Performance of different polyolefin composite modifiers.

Properties	PCM-H	PCM-1	PCM-2 ^1^	Technical Requirement
ash content/wt%	0.2	0.5	0.7	≤1.0
melt flow index/(g/10 min)	2.79	3.66	-	≥2.0
density/(g/cm^3^)	1.01	0.99	0.97	0.96~1.02
tensile strength/MPa	5.32	4.51	-	≥3.0
300% tensile strength/MPa	2.95	1.92	-	≥0.5
elongation at break/(g/cm^3^)	1357	1124	-	≥800

^1^ PCM-2 lacks fluidity due to crosslinking and agglomeration, making it impossible to measure its melt index, 300% tensile strength, tensile strength, and elongation at break.

**Table 4:** The relative molecular weight distributions.

Specimen	Mn	Mw	Mz	M_z+1_	PDI
SBS MA	781	2695	9474	17,884	3.45
PCM-1 MA	669	2632	9803	17,862	3.93
PCM-2 MA	714	8293	171,826	264,744	11.61
PCM-H MA	619	8140	1,719,973	288,284	13.15

**Table 5:** Comparison of basic properties of different polyolefin composite modified asphalts.

Properties		SBS MA	PCM-H MA	PCM-1 MA	PCM-2 MA	Technical Requirement
penetration (25 °C,100 g, 5 s) (0.1 mm)		50.1	44.5	42.6	38.6	≥40
softening point (°C)		80.7	103.5	100.6	95.6	≥80
ductility (5 cm/min, 5 °C) (cm)		28.9	36.5	34.5	34.0	≥30
dynamic viscosity (60 °C) (Pa·s)		25,619	2,846,151	2,151,612	1,477,000	≥50,000
Brookfield viscosity (175 °C) (Pa·s)		0.44	2.45	2.25	1.50	-
after TFOT	penetration ratio (25 °C) (%)	81.9	90.8	87.1	85.6	≥65
	ductility (5 cm/min, 5 °C) (cm)	16.8	30.2	26.6	25.8	≥20

**Table 6:** High-temperature performance grading results of SBS MA and different polyolefin composite modified asphalts.

Asphalt Type		G*·sinδ^−1^/kPa							High-Temperature Grade/°C
		58 °C	64 °C	70 °C	76 °C	82 °C	88 °C	94 °C	
SBS MA	original asphalt	8.37	4.13	2.74	1.62	1.05	0.68	-	PG 76
	residual asphalt	18.07	9.42	5.08	2.82	1.59	-	-	
PCM-H MA	original asphalt	44.03	25.76	15.22	9.45	6.21	4.25	2.71	PG 94+
	residual asphalt	47.35	29.47	18.17	11.18	6.90	4.29	2.99	
PCM-1 MA	original asphalt	42.69	25.16	14.35	8.51	5.53	3.87	2.75	PG 94+
	residual asphalt	43.59	25.72	15.66	9.68	6.18	4.08	2.88	
PCM-2 MA	original asphalt	34.63	19.81	11.54	7.12	4.72	3.28	2.33	PG 88
	residual asphalt	39.01	22.13	12.51	7.11	4.10	2.41	1.44	

## Data Availability

The raw data supporting the conclusions of this article will be made available by the authors on request.
